# The Multiscale Heterogeneity of Adipose Tissue in Health and Disease

**DOI:** 10.34133/research.1261

**Published:** 2026-04-22

**Authors:** Baile Wang, Xue Jiang, Qin Wang, Aimin Xu

**Affiliations:** ^1^ State Key Laboratory of Pharmaceutical Biotechnology, The University of Hong Kong, Hong Kong, China.; ^2^ Department of Medicine, The University of Hong Kong, Hong Kong, China.; ^3^ Guangdong-Hong Kong Joint Laboratory for Metabolic Medicine, The University of Hong Kong, Hong Kong, China.

## Abstract

Adipose tissue has evolved from a passive lipid store to a dynamic, heterogeneous endocrine organ, whose dysfunction is closely linked to a cluster of chronic metabolic diseases. While early studies focused on overall adiposity, emerging evidence demonstrates that depot-specific adipose tissue traits outperform body mass index alone in predicting various disease risks. Adipose tissue heterogeneity refers to the inherent differences across distinct adipose depots, which manifest at anatomical, cellular, and molecular levels, alongside functional specialization. Moving beyond descriptive characterization, this review proposes a multiscale, functionally grounded framework that translates adipose heterogeneity into precision risk management and depot-targeted therapies. We provide a holistic update to cover previously underappreciated adipose depots (perivascular, epicardial, and bone marrow adipose tissues) and integrate recent single-cell sequencing discoveries of novel cell subsets in adipose tissues. We systematically summarize the core hallmarks of adipose heterogeneity and depot-specific roles in health and disease, while assessing the strength of evidence linking cellular subsets to functional outcomes. We also discuss how emerging technologies such as spatial transcriptomics, organoid technology, and artificial intelligence-driven imaging analysis resolve pathogenic niches and build translatable risk models. Finally, we propose a translational framework to overcome key bottlenecks from preclinical validation to clinical implementation, aiming to advance personalized management of diseases related to adipose tissue dysfunction.

## Introduction

Adipose tissue is distributed throughout most human tissues except the brain, and its role has evolved dramatically from a historically perceived passive lipid storage depot to a dynamic, heterogeneous, and multifunctional endocrine organ with profound impacts on systemic health and disease [1]. Dysfunction, absence, or ectopic accumulation of adipose tissue results in a series of metabolic diseases, including obesity, type 2 diabetes (T2D), hyperlipidemia, cardiovascular disease (CVD), and metabolic dysfunction-associated steatotic liver disease (MASLD) [2]. This association between adiposity and disease traces back over 2,000 years to Hippocrates, and modern epidemiologic studies confirm that excess fat mass markedly correlates with a higher incidence of metabolic diseases [3].

Early investigations primarily focused on the association between overall adiposity and metabolic diseases. However, a growing body of evidence indicates that the quantity and functional properties of adipose depots at distinct anatomical sites exert differential effects on the onset and progression of various diseases. Recent epidemiologic studies based on large-scale magnetic resonance imaging (MRI) datasets (such as the UK Biobank) demonstrate that the precise distribution, quantity, and quality of adipose tissues outperform body mass index (BMI) in predicting multiorgan disease risk. A study of 6,716 individuals with obesity demonstrated that adipose tissue distribution differentially influences cardiometabolic risk [4]. Visceral adipose tissue (VAT) positively correlates with both CVD and T2D, while subcutaneous adipose tissue (SAT) is inversely associated with both [4]. Complementary research analyzing MRI images of 40,174 participants further confirms that specific body fat distribution patterns, rather than total adiposity, link to distinct cardiometabolic risk profiles [5]. For instance, a high-VAT and low-SAT profile increases CVD risk and T2D susceptibility [5]. All these findings highlight the clinical significance of adipose depot heterogeneity, explaining why BMI alone is insufficient to serve as a comprehensive predictor of cardiometabolic risk.

The concept of adipose tissue heterogeneity encapsulates the inherent differences in anatomical location, cellular diversity, and molecular signatures across distinct fat depots, which collectively determine their functional specialization and pathogenic potential [6]. This heterogeneity is not a static trait but a dynamic property that remodels in response to nutritional changes, environmental cues, metabolic status, and aging, thereby influencing the development and progression of a spectrum of closely related cardiometabolic diseases [7]. Beyond depot-level differences, cellular and lineage heterogeneity within these depots profoundly influences their function and explain why individuals respond differently to metabolic interventions. Distinct from prior reviews that have largely centered on the conventional subcutaneous–visceral fat dichotomy, our review broadens the focus to deliver an updated overview of underappreciated depots including perivascular adipose tissue (PVAT), epicardial adipose tissue (EAT), and bone marrow adipose tissue (BMAT), while incorporating recent breakthroughs such as the discovery of novel cell subpopulations in distinct adipose depots enabled by single-cell RNA sequencing (scRNA-seq). Furthermore, we dissect adipose tissue heterogeneity across anatomical, cellular, and molecular dimensions; elaborate on depot-specific characteristics and dynamic plasticity; discuss their multifaceted roles in health and disease; and evaluate the evidence linking specific cell subsets to functional outcomes. We also propose that understanding the spatial organization and microenvironmental niches within adipose tissue is key to reconciling the conflicting data and gaining deeper insights into pathogenic mechanisms. Additionally, we highlight how emerging technologies such as spatial transcriptomics, single-cell multiomics, organoid technology, and artificial intelligence (AI)-driven imaging analysis resolve pathogenic niches and build translatable risk models. We further propose a translational framework to overcome key bottlenecks, highlighting key steps from preclinical validation to clinical translation, thereby facilitating the development of personalized strategies for managing adipose tissue-related diseases.

## Anatomical Locations and Physiological Roles of Different Adipose Depots

**Fig. 1.**
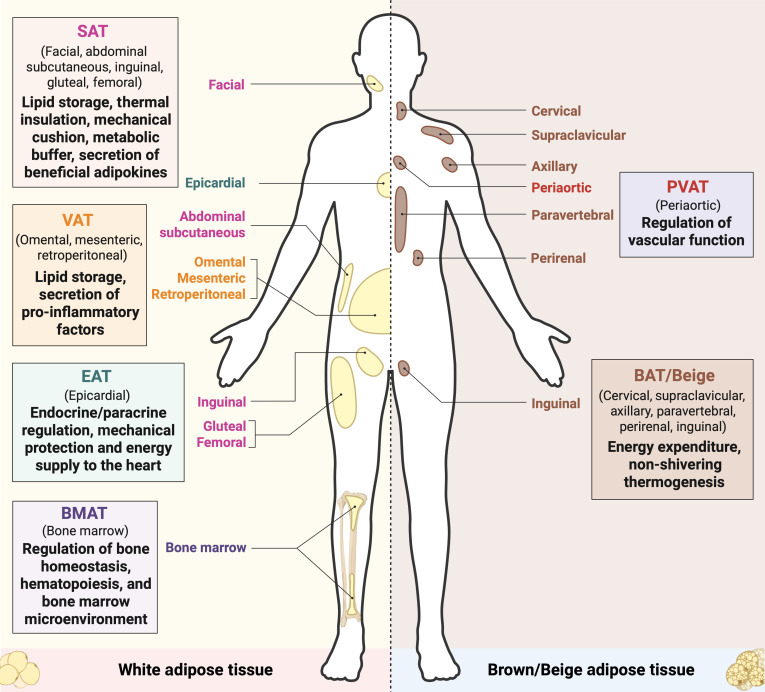
Anatomical locations and main functions of different adipose depots in humans. Schematic illustration of human adipose tissue distribution, showing white and brown/beige adipose tissues. Adipose depots are categorized into subcutaneous adipose tissue (SAT, under skin), visceral adipose tissue (VAT, around internal organs), and specialized types including brown/beige adipose tissue (BAT/beige, cervical, supraclavicular, axillary, paravertebral, perirenal, and inguinal regions), perivascular adipose tissue (PVAT, periaortic), epicardial adipose tissue (EAT, surrounding heart), and bone marrow adipose tissue (BMAT, within bone marrow cavity). Anatomical locations and main functions of each adipose depot are indicated. Illustration created using BioRender (https://BioRender.com).

Anatomical location is the most fundamental determinant of adipose tissue heterogeneity. Adipose tissues are strategically distributed throughout the body, forming distinct depots that can be broadly categorized into peripheral, visceral, and specialized niches. Peripheral depots, primarily SAT and brown adipose tissue (BAT), are located under the skin and in specific regions such as the cervical area, respectively (Fig. 1). VAT includes omental, mesenteric, retroperitoneal, and visceral fat surrounding internal organs, while specialized depots such as PVAT, EAT, and BMAT reside in unique anatomical contexts that enable tissue-specific crosstalk (Fig. 1) [6,8]. This spatial segregation is not arbitrary but is tightly linked to their embryonic origins, which contributes to their distinct functional properties. These depot-specific traits collectively modulate systemic metabolism and disease risk.

### Subcutaneous adipose tissue

SAT is the most abundant adipose depot in the body, accounting for ~80% of total fat mass in humans [9]. It is distributed throughout the subcutaneous layer, with prominent deposits in the abdomen, hips, and extremities. Embryonically derived from paraxial mesoderm, SAT is specialized for energy storage and is characterized by mature adipocytes containing a single large lipid droplet (unilocular) and low mitochondrial density [8]. It maintains systemic lipid homeostasis by storing excess nutrients as triglycerides within adipocytes and mobilizing them through regulated lipolysis during energy demand [10]. Beyond its storage function, SAT acts as a dynamic endocrine organ, secreting key adipokines such as adiponectin and leptin that modulate insulin sensitivity, appetite, and whole-body energy balance [11]. It also provides essential thermal insulation and mechanical cushioning to protect underlying tissues [12]. Adaptive expansion of SAT during energy surplus acts as a metabolic buffer that protects other organs from lipotoxicity caused by lipid overflow and ectopic fat deposition [13]. SAT is a key regulator of metabolic health mainly through its secretion of adiponectin, the most abundant adipokine in human serum. Adiponectin exerts insulin-sensitizing effects in muscle and liver by activating adenosine 5'-monophosphate (AMP)-activated protein kinase (AMPK) and peroxisome proliferator-activated receptor α (PPARα) signaling pathways [14], and is shown to alleviate both alcoholic and nonalcoholic fatty liver diseases in mice [15]. Clinical evidence supports the protective role of SAT by demonstrating that individuals with preferential SAT accumulation have a lower risk of obesity-induced metabolic dysregulation and enhanced insulin sensitivity compared to those with central obesity [16].

### Visceral adipose tissue

VAT, consisting of omental, mesenteric, retroperitoneal, and visceral fat surrounding the abdominal organs, accounts for 10% to 20% of total fat mass in men and 5% to 8% in women [17]. Derived from lateral plate mesoderm, VAT has distinct functional properties compared to SAT [8]. It demonstrates higher lipolytic sensitivity, lower insulin sensitivity, and a more proinflammatory secretome compared to SAT [18]. Under physiological conditions, VAT acts as a readily available energy source, releasing free fatty acids (FFAs) to meet systemic demands. It also functions as an endocrine organ by secreting various adipokines and vasoactive factors that influence metabolic homeostasis [18]. Additionally, VAT provides structural cushioning to protect intra-abdominal organs from physical stress [12]. However, its expansion is strongly linked to insulin resistance, T2D, dyslipidemia, MASLD, and other cardiometabolic diseases [2].

### Brown adipose tissue

BAT is a specialized thermogenic depot that plays a critical role in nonshivering thermogenesis and energy homeostasis, despite its limited proportion of total adipose mass. Localized primarily in the interscapular (during infancy), cervical, and supraclavicular regions in humans, BAT originates from paraxial mesoderm and is composed of multilocular brown adipocytes with high mitochondrial density [19]. The thermogenic function of BAT is primarily driven by uncoupling protein 1 (UCP1), a mitochondrial protein that uncouples oxidative phosphorylation from ATP production, releasing energy as heat [20]. Complementing this, UCP1-independent mechanisms, including calcium futile cycling, creatine-dependent substrate cycling, and triacylglycerol futile cycling, have also been identified using UCP1-null mice, underscoring the diverse regulatory mechanisms and metabolic flexibility of BAT [21,22]. Notably, early studies using 18F-fluorodeoxyglucose positron emission tomography (PET) scans confirmed the presence of functional thermogenic adipose tissue in adults, challenging the long-held belief that BAT is active only in infants [23].

### Beige adipose tissue

Beiging refers to the generation of thermogenic beige adipocytes within white adipose tissue (WAT). It primarily occurs within SAT and is a critical adaptive response that enhances energy expenditure and improves metabolic health. Beige adipocytes are multilocular and contain a moderate number of mitochondria. Cold adaptation is the most well-characterized inducer of beige adipocyte formation. Studies in mice have shown that cold exposure enhances the sympathetic outflow to SAT, releasing norepinephrine to trigger the β3-adrenergic receptor signaling cascade and thus promoting beiging of SAT [24]. Type 2 immunity also plays a critical role in beige adipocyte biogenesis, serving as a key bridge between immune homeostasis and metabolic adaptation. This immune-metabolic crosstalk is primarily mediated by a network of type 2 immune cells, including eosinophils, alternatively activated (M2) macrophages, and regulatory T cells (Tregs), which coordinate to promote beige adipogenesis and sustain thermogenic function. Central to this process is the secretion of type 2 cytokines, particularly interleukin-33 (IL-33), IL-4, and IL-13, which act as upstream activators of beige adipocyte differentiation. Upon activation by IL-33, group 2 innate lymphoid cells (ILC2s) secrete methionine-enkephalin peptides to induce eosinophil-derived IL-4 and IL-13, which in turn trigger M2 macrophage polarization, thus stimulating UCP1 expression and beige adipocyte differentiation [25–27]. The recruitment and accumulation of eosinophils and M2 macrophages induced by adipose-derived fibroblast growth factor 21 (FGF21) or adiponectin further support beiging via anti-inflammatory cytokines [28,29]. Tregs also promote beiging and thermogenesis in SAT by suppressing the expression of M1 macrophage markers (*Tnfa*, *Il6*, *iNos*, and *Ip10*) and enhancing the expression of M2 macrophage markers (*Mrc1*, *Arg1*, and *Il10*), thus slanting M2 macrophage polarization [30]. In addition to the immunoregulation, beige adipocytes can also transdifferentiate from preexisting lipogenic adipocytes, which originate from amphiregulin cells [31]. Exercise is another potent inducer of beige adipocyte activation, acting through multiple mechanisms including increased muscle-derived myokines, enhanced blood flow, and increased energy demand [32].

### Perivascular adipose tissue

PVAT is a specialized visceral adipose depot surrounding blood vessels, which regulates vascular homeostasis through paracrine signaling. It is present in most arteries, from large elastic arteries to small resistance vessels, and its function is tightly linked to vascular health [33]. Studies in mice have demonstrated that the PVAT surrounding the thoracic aorta displays brown-like thermogenic properties, characterized by high mitochondrial content and UCP1 expression [34]. Furthermore, PVAT functions as a critical vascular gatekeeper, exerting protective effects against vascular inflammation and atherosclerosis via UCP1-dependent anti-inflammatory pathways in mice and pigs [35]. Under physiological conditions, PVAT secretes a range of bioactive molecules, including adipokines (e.g., leptin, adiponectin, and omentin), cytokines/chemokines (e.g., IL-6, tumor necrosis factor-α [TNF-α], and monocyte chemoattractant protein-1 [MCP-1]), gaseous molecules (e.g., nitric oxide), and vasoactive substances, which collectively regulate vascular tone and homeostasis [36].

### Epicardial adipose tissue

EAT is a metabolically active adipose depot located between the myocardium and the visceral pericardium. It is a unique visceral depot that directly interacts with the heart. Under normal conditions, EAT provides mechanical protection and energy supply to the heart. It supplies FFAs to cardiomyocytes while buffering the heart from fluctuations in systemic FFA levels, as supported by both preclinical and clinical evidence [37]. Mechanistically, clinical observations using intravascular ultrasound have demonstrated that EAT absorbs shear and torsional stress generated by cardiac motion, thereby protecting the coronary vasculature [38]. Additionally, EAT is recognized to possess significant endocrine and paracrine activity, secreting multiple bioactive molecules including protective adipokines (e.g., adiponectin) and vasoactive factors (e.g., adrenomedullin) that modulate cardiac function [39]. These secretions are instrumental in maintaining coronary microvascular homeostasis, exerting anti-inflammatory effects, and supporting overall myocardial metabolic health.

### Bone marrow adipose tissue

BMAT is a specialized adipose depot embedded within the bone marrow cavity, accounting for up to 70% of bone marrow volume and representing over 10% of total fat mass in healthy adults [40]. Unlike other adipose depots, BMAT has a unique developmental origin, arising from bone marrow mesenchymal stem cells that share a common precursor with osteoblasts [41]. This shared lineage creates a competitive relationship, i.e., BMAT expansion is often accompanied by fewer osteoblasts and reduced bone formation, a phenomenon observed in aging, osteoporosis, and obesity in mice and humans [42,43]. In murine studies, BMAT has been classified into 2 subtypes, namely, regulated BMAT and constitutive BMAT, which differ transcriptomically from adipocytes in other fat depots [44]. Constitutive BMAT maintains stable lipid storage while regulated BMAT responds to metabolic changes [44]. Functionally, BMAT regulates hematopoiesis by secreting cytokines such as stem cell factor (SCF) and granulocyte-colony stimulating factor (G-CSF), which modulate hematopoietic stem cell (HSC) proliferation and differentiation [45]. BMAT also acts as a reservoir for FFAs, releasing them during energy deprivation to support bone and hematopoietic cell metabolism [46]. In glucose metabolism, BMAT is functionally distinct from WAT and BAT, with reduced insulin responsiveness and resistance to insulin- and cold-stimulated glucose uptake in both mice and humans, as shown by transcriptomic analyses and PET/computed tomography (PET/CT) [40]. Basal glucose uptake in human BMAT is greater than in axial bone, SAT, and even skeletal muscle, which emphasizes its underappreciated impact on systemic glucose homeostasis [40].

## Cellular Heterogeneity of Adipose Tissue

**Fig. 2.**
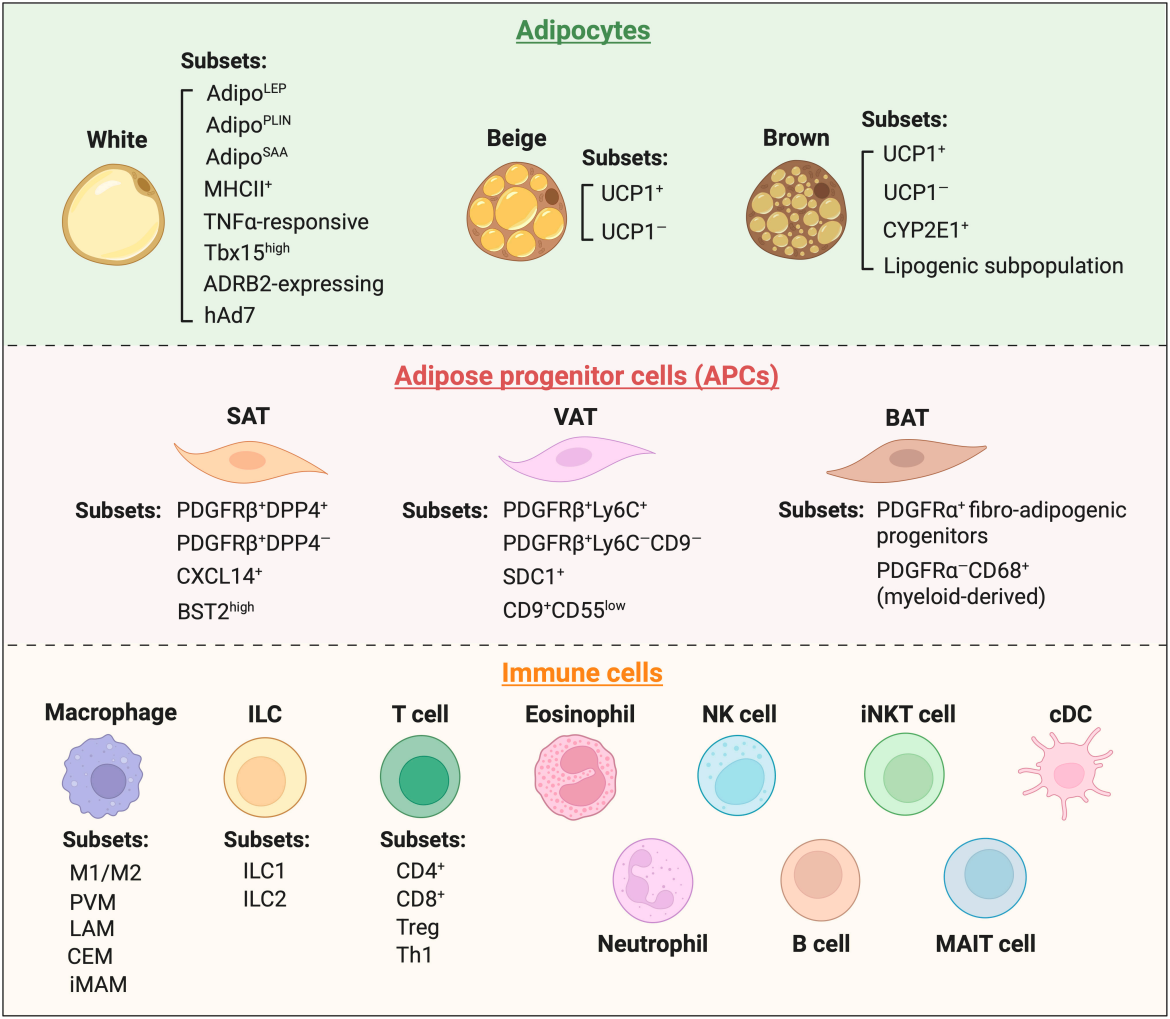
Cellular heterogeneity of adipose tissue. Categories and illustration of major cell types and subsets in adipose tissue. Adipocyte types differ between white adipose tissue (WAT) and BAT/beige fat, while adipose progenitor cells (APCs) exhibit depot-specific heterogeneity across SAT, VAT, and BAT. Immune cell composition varies dynamically with the physiological or pathological status of each adipose depot, with anti-inflammatory immune cells (M2 macrophages, conventional dendritic cells [cDCs], group 2 innate lymphoid cells [ILC2s], invariant natural killer T [iNKT] cells, Tregs, eosinophils, etc.) in SAT under lean conditions and proinflammatory immune cells (M1 macrophages, neutrophils, Th1 effector T cells, ILC1s, B cells, etc.) in VAT under obesogenic conditions. Illustration created using BioRender (https://BioRender.com).

Cellular diversity further amplifies adipose tissue heterogeneity beyond anatomical boundaries, with functional implications supported by single-cell or spatial transcriptomic profiling and complementary functional assays. Mature adipocytes constitute ~90% of the adipose tissue volume but only 15% to 30% of total cells. The remaining cellular component, termed the stromal vascular fractions (SVFs), harbors a rich repertoire of cells, including adipose progenitor cells (APCs), endothelial cells, fibroblasts, and a diverse array of immune cells such as macrophages, ILCs, T cells, and eosinophils [47]. Recent advances in spatial transcriptomics and single-cell analyses have identified distinct subpopulations of APCs, adipocytes, and immune cells, which further highlight the cellular diversity and complexity of adipose tissue (Fig. 2). However, a critical integration is still required to evaluate the strength of evidence linking cellular diversity to functional outcomes. To date, links between specific subpopulations and depot-specific physiology are largely supported by functional validation in murine models, but whether these observations can be translated into clinical relevance remains unknown. Therefore, future research should prioritize functional validation in human-based models to translate cellular mapping into clinical implications.

### Adipose progenitor cells

APCs, located within the SVFs, are a heterogeneous population of progenitor cells that maintain adipose tissue homeostasis and mediate remodeling, with functional specialization shaped by depot location, sex, disease state, and microenvironmental cues. Genetic lineage tracing studies and scRNA-seq analysis have robustly characterized functionally distinct APC subpopulations across different anatomical depots, primarily in mouse models [48–55], providing a strong mechanistic foundation [56]. In mouse VAT, 2 well-defined subpopulations have been identified: fibro-inflammatory progenitors (FIPs) and adipogenic PDGFRβ-expressing perivascular cells [48]. FIPs (LY6C^+^PDGFRβ^+^ cells) exhibit strong proinflammatory, profibrotic, and antiadipogenic phenotypes, while the LY6C^−^CD9^−^PDGFRβ^+^ subpopulation highly expresses PPARγ and other proadipogenic genes, possessing robust adipogenic capacity [48,49]. In mouse SAT, a hierarchical APC system defined by dipeptidyl peptidase-4 (DPP4) expression has been clearly delineated, comprising multipotent DPP4^+^ APCs that mediate cold-induced thermogenic remodeling via IL-33 secretion and committed DPP4^−^ APCs that express high levels of proadipogenic PPARγ [50]. Further detailed comparative analysis of epididymal VAT and SAT in mice reveals depot-specific APC subpopulations with distinct adipogenic stages. SAT-specific BST2^high^ APCs differentiate into beige adipocytes upon cold exposure, while VAT-derived SDC1^+^ APCs and SAT-resident CXCL14^+^ APCs modulate fibrosis and anti-inflammation in obesity, respectively [51]. Unlike in VAT and SAT, APCs in classical BAT predominantly express myogenic factor 5 (Myf5), while PDGFRα^+^ fibro-adipogenic progenitors and myeloid-derived subsets (such as PDGFRα^−^CD68^+^) serve as additional brown adipocyte progenitors that contribute to BAT regeneration [52]. Mouse studies have also mechanistically demonstrated that sex shapes APC function, with male SAT progenitors exhibiting blunted adipogenic potential due to higher inhibitory PPARγ S112 phosphorylation [53]. Crucially, human studies provide direct translational insights, revealing that VAT APC heterogeneity is perturbed in T2D, with expanded CD9^+^CD55^low^ subsets disrupting glycemic control by forming a detrimental niche for lipolysis, thereby highlighting the pathological relevance and clinical significance of APC subpopulation imbalance [56]. The functional heterogeneity of APCs is also regulated by microenvironmental cues. For example, obesity-induced hypoxia in mouse VAT has been shown to promote the expansion of a proinflammatory APC subpopulation that secretes IL-8 [55], while cold exposure induces the proliferation of a thermogenic APC subpopulation in mouse SAT that differentiates into beige adipocytes [54]. Collectively, these findings, particularly those with mechanistic validation in mouse models, underscore how APC heterogeneity governs adipose tissue function and homeostasis, distinguishing well-validated core subsets (such as PDGFRβ^+^ and DPP4^+^) as possible translational targets from those that still require functional interrogation.

### Adipocytes

Adipocytes are the most functionally diverse cell type in adipose tissue, with 3 major subtypes: white, brown, and beige adipocytes. White adipocytes, the most abundant subtype, are unilocular with low mitochondrial density, specialized for lipid storage and adipokine secretion. Brown adipocytes are multilocular with high mitochondrial density, specialized for thermogenesis. Beige adipocytes are multilocular with moderate mitochondrial density and are inducible within SAT in response to cold exposure or pharmacological stimuli for energy dissipation. While brown and beige adipocytes are classically characterized as UCP1^+^ cells, recent studies in UCP1 knockout mice have demonstrated the existence of multiple UCP1-independent thermogenic mechanisms with significant implications for therapeutic targeting [21,22]. High-resolution scRNA-seq and single-nucleus RNA-seq (snRNA-seq) analyses have consistently uncovered distinct subpopulations within each adipocyte subtype, linking cellular diversity to tissue function and disease in both mouse and human tissues [57–65]. In BAT, mechanistically characterized subsets such as CYP2E1^+^ cells suppress thermogenesis under warm conditions [57], while lipogenic brown adipocytes surrounding UCP1^high^ cells mediate “thermogenic memory” through acylcarnitine production after cold exposure [58]. In mouse beige fat, both UCP1^+^ and UCP1^–^ populations have been shown to exhibit a robust futile creatine cycle, with even predominantly UCP1^–^ abdominal beige fat retaining clear thermogenic capacity [59].

White adipocyte subpopulations extend far beyond brown and beige adipocytes. While mouse WAT shows 3 to 5 subpopulations with minimal depot-specific differences, human WAT demonstrates greater diversity with 3 to 7 depot-specific subsets and limited cross-species conservation, suggesting human-specific functional nuances [60]. Among these, TNF-α-responsive type 2 adipocytes have been mechanistically demonstrated to regulate lipolysis via fat-specific protein 27 (FSP27) in a transgenic mouse model [61]. Other identified subsets include antigen-presenting MHCII^+^ adipocytes in mouse SAT [31], together with Tbx15^high^ and ADRB2-expressing cells implicated in glycolysis and metabolic health [62,63], though their functional significance is largely supported by observational and correlative data from mouse studies. In human SAT, high-resolution studies have robustly characterized 3 functionally distinct subtypes (Adipo^LEP^, Adipo^PLIN^, and Adipo^SAA^), with Adipo^PLIN^ definitively emerging as the primary insulin-responsive population [64], thus identifying a precise cellular target for improving insulin sensitivity in humans. Furthermore, a cross-species single-cell atlas has powerfully linked specific human adipocyte subtypes (such as hAd7) directly to T2D risk, providing strong, clinically relevant associations [65]. However, further functional perturbation studies in models that accurately recapitulate human adipocyte biology remain essential to establish the causal links between specific adipocyte subsets with metabolic disease traits.

### Immune cells

The immune cell component of adipose tissue is a major contributor to cellular heterogeneity, with distinct resident and recruited populations that act as central regulators of adipose tissue homeostasis, remodeling, and metabolic flexibility. Adipose tissue macrophages (ATMs) are the most abundant immune subset, comprising up to 50% of nonadipocyte cells in obese depots [66]. The shift from anti-inflammatory M2-like macrophages to proinflammatory M1-like macrophages during obesity is a well-established causal driver of insulin resistance and adipose tissue inflammation, primarily elucidated through mechanistic studies in mice [66,67]. Beyond the canonical M1- and M2-like macrophages, scRNA-seq studies have revealed further specialized ATM subpopulations in mouse adipose tissues, such as perivascular macrophages (PVM), lipid-associated macrophages (LAM), collagen-expressing macrophages (CEM), and inflammatory and metabolically activated macrophages (iMAM). These subsets of macrophages perform niche-specific functions, such as extracellular matrix (ECM) modulation and lipid metabolism buffering [68,69].

The adipose immune landscape also includes diverse innate immune cells (e.g., natural killer [NK] cells, ILCs, mucosal-associated invariant T [MAIT] cells, and eosinophils) and adaptive immune cells (e.g., CD4^+^ and CD8^+^ T cells, B cells, and Tregs), whose composition and activity are dynamically tuned by metabolic status [70,71]. In lean conditions, resident anti-inflammatory cells such as M2 macrophages, conventional dendritic cells (cDCs), ILC2s, invariant natural killer T (iNKT) cells, Tregs, and eosinophils maintain tissue homeostasis by secreting anti-inflammatory cytokines including IL-4, IL-10, and IL-13. For example, eosinophils drive M2 polarization and beige adipogenesis, while Tregs suppress inflammation and support adipogenic differentiation in mice [26,72]. In obesity, this landscape shifts dramatically, particularly in VAT, toward a proinflammatory state. This involves substantial ATM expansion, a dominance of proinflammatory CD11c^+^ (M1-like) macrophages, recruitment of neutrophils and Th1 effector T cells, a reduction in beneficial Tregs and IL-4-producing eosinophils, and induction of ATM aggregation into crown-like structures surrounding dying adipocytes [66,67,73–75]. While the causal contributions of these immune cells to obesity-associated adipose tissue inflammation have been established through loss- and gain-of-function studies in mice, the mechanisms by which obesity triggers sequential changes in the adipose immune landscape remains elusive. Furthermore, current research has largely focused on their local roles within the adipose niche, whereas their direct impacts on systemic insulin sensitivity and whole-body metabolic homeostasis remain poorly defined.

## Depot-Specific Molecular Signatures of Adipose Tissue

Molecular signatures, including depot-specific secretomes and metabolic fingerprints, represent the functional readout of adipose tissue heterogeneity. The secretome of adipose tissue, comprising adipokines, cytokines, and exosomal miRNAs, varies dramatically across depots. SAT is a major source of adiponectin (an insulin-sensitizing adipokine), while VAT secretes high levels of proinflammatory cytokines such as IL-6, a pattern well-documented in human studies through direct sampling of distinct fat depots [76]. Metabolic fingerprints, defined by parameters such as lipolytic capacity, mitochondrial density, and expression of thermogenic proteins, further distinguish depots into white and brown/beige adipose tissue. These molecular differences are not only inherent but also dynamically regulated by physiological and pathological stimuli, underscoring the plastic nature of adipose tissue heterogeneity [77].

### Secretome

The secretome of adipose tissue is a complex mixture of bioactive molecules, which is not a simple sum of cellular outputs but arises from paracrine crosstalk between multiple cell types, including adipocytes, APCs, microvascular endothelial cells, and immune cells, a concept established through in vitro studies using human adipose tissue-derived cells [78]. Adipose tissue exhibits pronounced depot-specific secretome heterogeneity that shapes its functional specialization. In humans, VAT displays inherently higher secretory capacity than SAT, releasing greater amounts of proinflammatory cytokines (TNF-α, IL-6, and IL-1β), vascular endothelial growth factor, and plasminogen activator inhibitor 1, alongside angiogenic factors that support its expandability and chemokines (such as MCP-1) that recruit macrophages/monocytes via IL-6/JAK signaling, thus reinforcing its proinflammatory and disease-promoting phenotype [79,80]. VAT also secretes high levels of leptin (disrupted by obesity-induced leptin resistance) but low levels of adiponectin, while SAT serves as the primary source of the insulin-sensitizing adiponectin and releases anti-inflammatory cytokines (IL-10 and transforming growth factor-β [TGF-β]) and protective factors such as vitamin D-binding protein. These depot-specific secretory patterns have been corroborated in human cohort studies [81] and further supported by proteomic analyses in rodent models [82]. Despite the pronounced depot-specific differences, protein secretion from both VAT and SAT is completely refractory to endurance exercise training, as demonstrated in a controlled mouse study [83], indicating that these signatures are intrinsically programmed and may require more targeted interventions for modulation. Exosomal miRNAs are also key components of the secretome, which mediate intercellular communication between adipose tissue and other organs. The investigation of exosomal miRNAs is a rapidly evolving area with mechanistic data from cell and animal models, as well as human-derived correlative data. For example, SAT-derived exosomes contain miR-130b-3p, which targets PPARγ coactivator 1α (PGC-1α) to regulate apoptosis of rat ventricular cardiomyocytes [84]. In contrast, VAT-derived exosomes containing miR-34a or miR-27b-3p inhibit M2 macrophage polarization to promote obesity-induced adipose inflammation or induce endothelial dysfunction through PPARα suppression, respectively [85,86]. These mechanisms have been validated using knockout mouse models and are supported by clinical correlations in human cohorts. However, the precise roles and therapeutic potential of key exosomal miRNAs in humans remain to be fully established, highlighting technical difficulties in translating animal-based discovery to clinical application.

### Metabolic fingerprints

Metabolic fingerprints, defined by the intrinsic metabolic properties of adipose depots, are not only a key molecular signature of heterogeneity but also fundamental and well-characterized physiological differences with clear causal links to disease. The high lipolytic capacity of VAT is due to the abundant expression of β-adrenergic receptors and low expression of antilipolytic insulin receptors, a pattern initially characterized in human adipose tissue biopsies [87], which makes VAT a primary source of circulating FFAs, and a major contributor to hepatic steatosis and insulin resistance when it is overexpanded [88]. In contrast, human SAT has lower lipolytic capacity but a higher re-esterification rate, enabling efficient lipid storage [89]. The metabolic differences are tightly linked to the functional identity of each depot, with thermogenic depots (BAT and beige-enriched SAT) conferring metabolic protection and dysregulated storage depots (VAT) contributing to disease. These findings highlight well-defined causal mechanisms underlying such depot-specific traits, with thermogenic activation of BAT/beige and inhibition of VAT lipolysis emerging as translationally actionable strategies.

## Heterogeneous Roles of Different Adipose Depots in Health and Disease

**Fig. 3.**
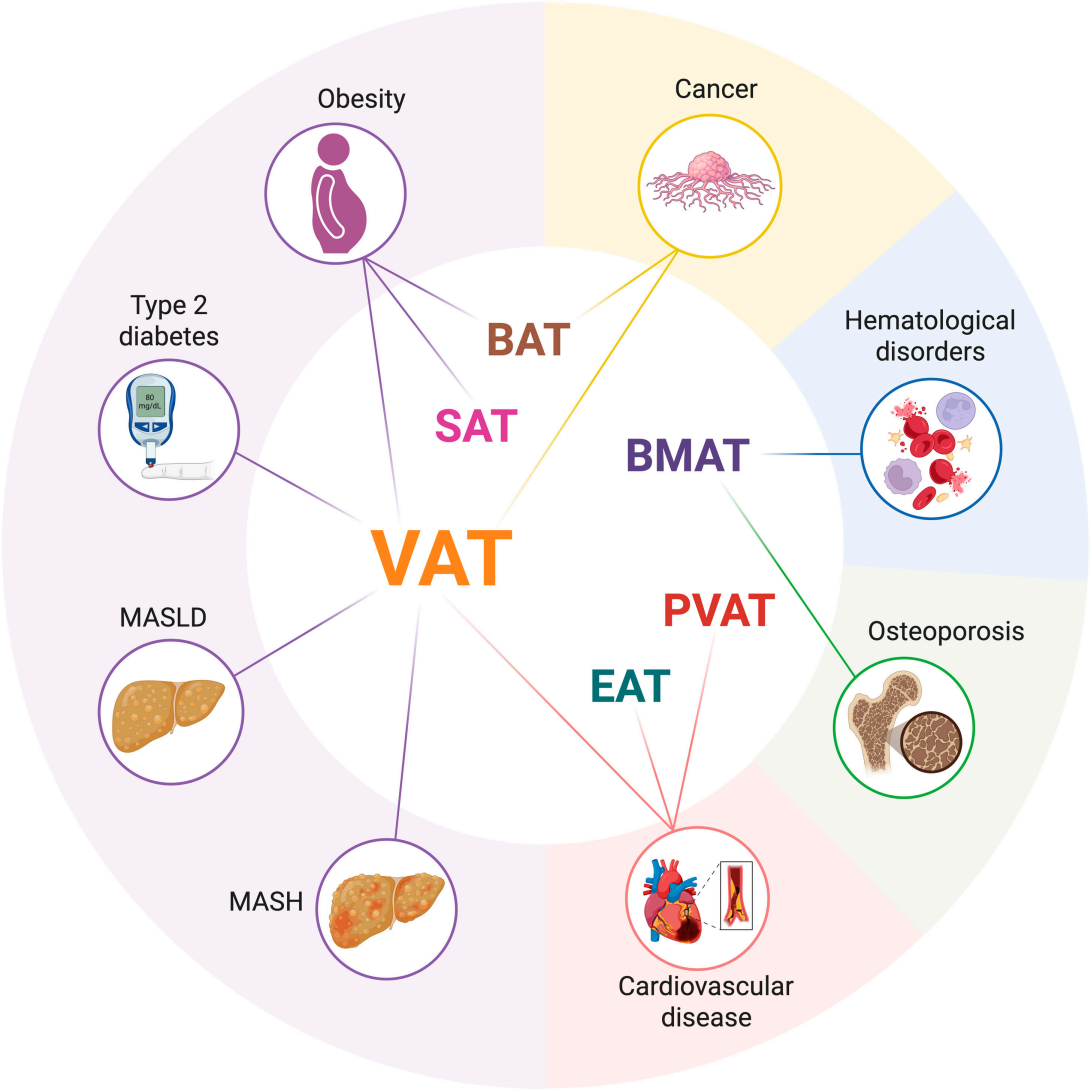
The association between different adipose depots and various diseases. VAT shows broad associations with obesity, type 2 diabetes, metabolic dysfunction-associated steatotic liver disease (MASLD), metabolic dysfunction-associated steatohepatitis (MASH), cardiovascular diseases, and even cancer. PVAT and EAT correlate primarily with cardiovascular disorders, while BMAT links to hematological disorders and metabolic bone diseases such as osteoporosis. SAT associates with pear-shaped obesity, while BAT exerts beneficial effects on obesity and certain cancers. Illustration created using BioRender (https://BioRender.com).

Adipose tissue heterogeneity shapes its diverse contributions to health and disease. VAT is intertwined with obesity and its related metabolic diseases, such as T2D, MASLD, and metabolic dysfunction-associated steatohepatitis (MASH). In contrast, SAT and BAT exert protective effects against obesity and its related metabolic disorders. PVAT and EAT depots are increasingly linked to cardiovascular disorders, while BMAT is closely associated with osteoporosis and hematological conditions. These depot-specific associations underscore the clinical significance of the functional diversity of adipose tissues (Fig. 3).

### The role of SAT in metabolic dysfunction during obesity

SAT not only serves as the body’s primary reservoir for energy storage, but also secretes adipokines and hormones that regulate metabolism, hunger, and satiety. When energy intake is excessive, SAT initially undergoes “healthy expansion” through adipocyte hypertrophy and hyperplasia with sufficient angiogenesis and preservation of endocrine function, thereby allowing surplus lipids to be safely sequestered within the subcutaneous depot [90]. However, the storage capacity of SAT is finite. With further progression of obesity, excessive adipocyte enlargement combined with inadequate vascular supply leads to local hypoxia and endoplasmic reticulum stress, which in turn activate inflammatory pathways and promote macrophage infiltration, establishing a chronic inflammatory positive feedback loop [91,92]. Inflammatory mediators such as TNF-α and IL-6 further impair adipocyte differentiation and enhance lipolysis, ultimately resulting in a collapse of the lipid storage capacity of SAT [93–95]. Consequently, excess energy “spills over” in the form of FFAs into VAT and ectopic sites such as the liver, myocardium, and skeletal muscle, where it induces pronounced systemic insulin resistance through direct lipotoxicity and persistent inflammatory signaling, thereby contributing to hepatic steatosis, myocardial dysfunction, and CVD [94,96–98].

In parallel, the endocrine profile of SAT undergoes a profound shift during obesity development. The protective adipokine adiponectin is markedly down-regulated, whereas proinflammatory cytokines and leptin are abundantly secreted, transforming SAT from a “metabolic protector” into an “inflammatory driver” [77,99]. This aberrant secretory profile directly promotes systemic insulin resistance, endothelial dysfunction, and chronic low-grade inflammation, positioning SAT as a hub linking obesity to metabolic syndrome. Large-scale imaging studies such as CT and MRI have demonstrated that, at comparable BMI levels, individuals with relatively greater SAT but lower visceral and ectopic fat tend to exhibit a more favorable metabolic phenotype, whereas those with limited SAT capacity or SAT dysfunction are more prone to develop hepatic steatosis, myocardial fat accumulation, and intramuscular fat deposition [13,100–103]. Thus, SAT acts as a critical metabolic buffer, whose dysfunction (marked by inflammatory transformation and lipid spillover) is an important contributor to systemic insulin resistance and cardiometabolic disease.

### VAT dysfunction in obesity-related metabolic disorders

Excess calorie intake triggers VAT expansion primarily through adipocyte hypertrophy, accompanied by limited hyperplasia. In mouse models, this expansion is supported by ECM remodeling and angiogenesis, which are initially driven by mild proinflammatory signals from resident macrophages to facilitate tissue plasticity [104]. However, once adaptive capacity is exhausted, hypertrophic adipocytes develop endoplasmic reticulum stress, activate NF-κB, and secrete proinflammatory mediators such as IL-6 to initiate VAT inflammation, as evidenced by human ex vivo studies and rodent models [105]. Regional hypoxia resulting from inadequate angiogenesis in expanding VAT further amplifies this response via hypoxia-inducible factor 1α (HIF1α), promoting the secretion of chemokines and cytokines that activate or attract immune cells, as initially shown in mouse studies and corroborated by human adipose tissue analyses [106].

Structural damage further exacerbates VAT inflammation. Apoptotic/necroptotic adipocytes release damage-associated molecular patterns such as HMGB1, while senescent adipocytes, macrophages, and endothelial cells exhibit a senescence-associated secretory phenotype rich in proinflammatory factors and microRNAs, a conceptual framework established in mouse models and extended to human aging and obesity [107,108]. These signals recruit circulating immune cells, including monocytes which differentiate into proinflammatory M1 macrophages, T cells, B cells, ILC1s, neutrophils, and mast cells, all of which outnumber resident populations and form a chronic inflammatory microenvironment [109]. Macrophages aggregate into crown-like structures around dead adipocytes, secreting proinflammatory cytokines and chemokines that amplify inflammation, while neutrophils release elastase and extracellular traps to impair insulin signaling [73,110,111]. Concurrently, ECM dysregulation leads to collagen accumulation and endotrophin release, further enhancing proinflammatory reactivity, as demonstrated in mouse studies [112]. Notably, while this inflammatory cascade initially aims to restore homeostasis, the threshold at which it transitions to detrimental effects remains undefined, and the role of senescent cells in supporting or impairing tissue function requires further investigation [113].

Anatomically, human VAT drains directly into the portal vein, delivering excess FFAs to the liver, which promotes hepatic steatosis and insulin resistance [114]. This process is a key initiating factor in the development of MASLD and liver-derived hyperglycemia [115]. In parallel, the proinflammatory cytokines (e.g., TNF-α and IL-6) and vasoactive substances such as angiotensinogen produced by hypertrophied VAT collectively contribute to insulin resistance, hypertension, and endothelial impairment [18]. Clinically, visceral obesity exhibits a strong association with all recognized components of metabolic syndrome, including dyslipidemia, hypertension, and hyperglycemia, as evidenced by large-scale epidemiological cohort studies [101,116]. This condition substantially elevates the risk of developing T2D, coronary artery disease (CAD), and certain malignancies. Collectively, VAT is a primary inflammatory driver in obesity. Characterized by immune cell infiltration and an altered secretory profile, VAT dysfunction represents a dynamic process that directly promotes hepatic insulin resistance and contributes to metabolic syndrome. Nonetheless, the precise triggers of its pathological transition and its context-dependent effects across obesity subtypes remain poorly defined.

### Roles of PVAT and EAT dysfunction in CVD

Healthy PVAT directly safeguards vascular function by suppressing local inflammation, releasing protective adipokines and vasodilating factors to modulate vascular tone. However, under pathological conditions such as obesity, aging, or metabolic syndrome, PVAT undergoes phenotypic “whitening” and structural remodeling. It actively contributes to aortic stiffness and maladaptive arterial remodeling through various mechanisms. Dysfunctional PVAT secretes excessive reactive oxygen species (ROS) and proinflammatory cytokines, which contribute to vascular stiffening and impaired vascular function [117]. Notably, PVAT from hypertensive patients exhibits up-regulated expression of lysyl oxidase, an enzyme that crosslinks collagen fibers, further enhancing arterial stiffness [118]. Additionally, PVAT influences vascular ECM remodeling by secreting matrix metalloproteinases (MMPs) and tissue inhibitor of metalloproteinases (TIMPs) in rodent models [119]. In obesity, PVAT exhibits elevated MMP-2 and MMP-9 activity, which degrades elastin and facilitates collagen deposition, thereby contributing to increased arterial stiffness and adverse vascular remodeling [120]. Furthermore, PVAT communicates with the vascular wall through adipokine signaling and extracellular vesicles. PVAT-derived exosomes from obese mice contain increased miR-145 that promotes vascular smooth muscle cell (VSMC) proliferation and migration [121]. As demonstrated by in vitro and animal studies, PVAT-derived leptin stimulates VSMC hypertrophy, contributing to vascular wall thickening [122]. Clinical studies have also shown that PVAT thickness correlates with arterial stiffness and CVD risk, highlighting its role as a modulator of vascular disease [123]. Thus, dysfunctional PVAT acts as a critical mediator in the “outside-in” progression of CVD, linking systemic metabolic dysfunction to localized vascular injury.

EAT expansion in obesity and CAD exerts detrimental effects via 3 overarching mechanisms: direct paracrine/vasocrine diffusion of harmful mediators into the myocardium and coronaries, local and systemic inflammatory crosstalk, and mechanical burden on the heart. Human histological and imaging studies have shown that EAT expands and becomes inflamed in obesity and CAD [124]. Its volume increases and it becomes infiltrated by proinflammatory immune cells, especially M1 macrophages. Harmful mediators including excess FFAs and ROS diffuse directly into the adjacent myocardium and coronary arteries due to the absence of a fascial barrier, promoting endothelial dysfunction, oxidative stress, and atherosclerotic plaque instability [125]. Excessive FFAs from EAT also contribute to myocardial lipotoxicity, insulin resistance, and fibrotic remodeling [126]. Moreover, increased EAT imposes a mechanical burden on the heart, exacerbating ventricular hypertrophy and diastolic impairment [127]. The inflammatory crosstalk between EAT and the adjacent myocardium plays a vital role in the development and progression of heart failure (HF), occurring through multiple pathways that create a vicious cycle of myocardial dysfunction and metabolic derangement, thereby exacerbating HF progression. Dysfunctional EAT secretes a plethora of proinflammatory adipokines and cytokines that directly infiltrate the cardiac muscle through paracrine and vasocrine pathways. In patients with HF with preserved ejection fraction, EAT exhibits markedly elevated expression levels of proinflammatory cytokines, including IL-1β, IL-6, and TNF-α, demonstrating a strong correlation with echocardiographic indices of diastolic dysfunction [128]. In response to this inflammatory milieu, mouse myocardial tissue up-regulates chemokines such as MCP-1, which facilitates the recruitment of macrophages into EAT, thereby establishing a self-perpetuating inflammatory feedback loop that exacerbates myocardial pathology [129]. In cardiomyocytes, TNF-α impairs calcium handling and reduces contractility via activation of the NF-κB pathway [130], while IL-1β facilitates cardiomyocyte hypertrophy and apoptosis via activation of the p38 mitogen-activated protein kinase pathway, concurrently contributing to endothelial dysfunction within the coronary microvasculature [131]. Notably, EAT from patients with HF exhibits dramatic up-regulation of inflammasome components, particularly NLRP3, which enhances the processing and release of IL-1β [132]. This localized inflammatory environment is further intensified by a decrease in the secretion of protective adipokines such as adiponectin, which typically exert anti-inflammatory and cardioprotective effects [133]. The inflammatory interplay extends beyond cytokines to encompass extracellular vesicles. Exosomes derived from EAT in HF patients are enriched with specific microRNAs (such as miR-27a, miR-130a, and miR-200c) that facilitate myocardial fibrosis by activating TGF-β signaling pathways in cardiac fibroblasts [134]. These exosomes also carry dysfunctional mitochondrial DNA that activates Toll-like receptor 9 (TLR9)-mediated inflammatory responses in mouse cardiomyocytes [135]. In coronary atherosclerosis, EAT-derived exosomal miRNAs mediate intercellular communication with coronary vasculature to regulate plaque formation [136]. For example, EAT-derived miR-92a-3p improves the myocardial redox state by modulating Wnt5a signaling and is related to improved clinical outcomes [137]. Clinically, a study on CAD patients identified 385 differentially expressed proteins in EAT compared to non-CAD controls, with enrichment in pathways related to lipid metabolism, inflammation, and oxidative stress, providing a human proteomic landscape of EAT dysfunction [138]. These changes contribute to myocardial inflammation, fibrosis, and ischemia, linking EAT dysfunction to adverse cardiac outcomes [139]. In summary, converging evidence from human studies and animal models positions dysfunctional EAT as a central endocrine and inflammatory organ that exacerbates cardiac pathology through multifaceted crosstalk, making it a compelling target for cardiometabolic intervention.

### Pleiotropic roles of BMAT in bone homeostasis and hematopoiesis

BMAT expands with aging, obesity, or anorexia nervosa, which is far from a passive niche filler but a multifaceted regulator of bone homeostasis and hematopoiesis. Recent preclinical and clinical studies have linked BMAT dysfunction to bone fragility and hematological disorders, highlighting its role in skeletal and hematopoietic health [140]. Its relationship with skeletal health is notably complex. While BMAT correlates inversely with trabecular bone loss in older women [141] and bone mass in age-related osteoporosis [142], positive associations are observed in early-pubertal girls [143], with gender, age, and metabolic status shaping these dynamics. Anorexia nervosa and caloric restriction paradoxically increase BMAT alongside osteoporosis [144,145], highlighting context-dependent effects that extend beyond simple causality.

In addition to bone homeostasis, BMAT also exerts dual roles in hematopoiesis. Irradiation- or obesity-induced BMAT expansion impairs hematopoietic recovery in mice [146,147]. Conversely, BMAT supports myelopoiesis by releasing lipolytic fatty acids and paracrine factors such as SCF in mice with energy deficit and hematopoietic regeneration [148,149], underscoring its role as both an energy reservoir and a cytokine source for hematopoietic homeostasis. Adiponectin from BMAT enhances quiescence exit of HSCs and hematopoietic recovery [150], though conflicting findings arise from different mouse models [151]. Collectively, BMAT is a context-dependent, pleiotropic regulator of bone and hematopoietic homeostasis. This functional duality (capable of both supporting and impairing tissue homeostasis) underscores the need for BMAT-specific models to resolve conflicting observations and to elucidate its underlying mechanisms, which is critical for understanding its roles in age- and obesity-related diseases.

### Beneficial effects of BAT and beige fat on metabolic health

BAT and beige fat exert profound beneficial effects on metabolic health by enhancing whole-body energy expenditure (although the magnitude is generally considered more modest and variable in humans than in small animals), clearing glucose, triglycerides, and branched-chain amino acids (BCAAs), and improving insulin sensitivity [152,153]. While robust data from mouse models such as BAT transplantation in obese mice support the causal roles in reversing weight gain and obesity-induced insulin resistance [154], the functional relevance of thermogenic fat, particularly beige fat, in adult humans remains an area of active debate. Human studies using PET/CT have shown a close association of elevated BAT activity with lower BMI, improved insulin sensitivity, and reduced risk of T2D and dyslipidemia, but these are largely correlational findings [155]. Establishing causality and quantifying the precise metabolic impact in humans is challenging due to inherent limitations in assessment methods and the heterogeneous nature of human populations. This highlights a key obstacle in translating promising preclinical findings. For instance, beige fat provides beneficial lipids such as phosphatidylserine that improves mitochondrial function and activates thermogenesis in SAT to potentiate the anti-obesity effect of ketogenic diets in mice [156]; however, whether and to what extent these mouse-based findings on beige fat functions can be translated to human physiology constitutes a major research gap.

A growing body of evidence suggests that pharmacological interventions activating BAT and/or beige fat may be beneficial for obesity-related metabolic disorders. β3-adrenergic agonists (e.g., mirabegron), GLP-1 receptor agonists (e.g., liraglutide), and natural compounds (e.g., berberine and resveratrol) that activate BAT and promote beiging of SAT have been shown to ameliorate metabolic dysfunction in preclinical and clinical studies [157–159]. A randomized clinical trial of mirabegron found that 4 weeks of treatment increased BAT metabolic activity, energy expenditure, insulin sensitivity, and pancreatic β cell insulin secretion, with no obvious adverse effects [160]. However, given that none of these pharmacological agents is specific to brown/beige adipocytes, these studies cannot establish the causal relationship between brown/beige adipocyte activation and metabolic benefits. To overcome the current reliance on correlational human data and to fully elucidate the functional relevance of thermogenic fat, future research should focus on the identification of pharmacological tools that can specifically activate brown/beige adipocytes for interventional studies in humans as well as on the development of more precise and noninvasive methods for assessing thermogenic activity and brown/beige fat functions in humans. Consequently, activating thermogenic adipose tissue (BAT/beige) presents a promising, albeit complex, therapeutic avenue to enhance energy expenditure and substrate clearance, thereby ameliorating obesity-associated metabolic disorders.

## Translational Implications

### Adipose depot-specific biomarkers in metabolic diseases and CVDs

Distinct adipose depots have unique molecular signatures that reflect the pathogenesis of distinct metabolic disorders and CVD, providing promising avenues for precision diagnosis and therapeutic intervention. As demonstrated in prospective human cohort studies, the plasma leptin-to-adiponectin ratio can serve as an early biomarker to predict the development of T2D 3 to 5 years prior to clinical diagnosis [161]. This ratio reflects the imbalance between VAT-derived leptin and SAT-derived adiponectin, providing a composite measure of adipose tissue dysfunction. Fatty acid binding protein 4 (FABP4 or A-FABP) is a proinflammatory adipokine primarily secreted by VAT, which acts as a reliable prognostic biomarker for metabolic diseases and CVDs [162–164]. Elevated circulating FABP4 levels correlate with waist circumference, blood pressure, and dyslipidemia in cross-sectional human studies [162], and positively associate with cerebral infarct volume in patients with acute ischemic stroke [165]. Furthermore, circulating FABP4 level has been shown as an independent predictor for metabolic syndrome, T2D, and all-cause and cardiovascular mortality in T2D patients, based on longitudinal cohort studies with up to 10 years of follow-up [166–168]. Additionally, VAT-derived retinol binding protein 4 (RBP4) contributes to systemic insulin resistance, with its serum levels strongly correlating with glucose intolerance in human subjects [169]. The heart-surrounding EAT functions as both a biomarker and a driver of CVD through its secretion of proinflammatory adipokines. Elevated resistin levels in EAT are strongly linked to CAD and myocardial infarction in patients undergoing cardiac surgery, where it promotes endothelial dysfunction. These adipokines not only reflect local inflammation but also serve as measurable biomarkers for early cardiovascular risk detection and severity stratification [170]. The vasocrine and paracrine functions of PVAT place it as a direct regulator of vascular tone and health. Dickkopf-1 (DKK1), a canonical Wnt signaling inhibitor, is a critical novel biomarker from inflamed PVAT. Its secretion is stimulated by cytokines, and it promotes endothelial dysfunction and smooth muscle cell proliferation. Elevated plasma DKK1 levels are directly linked to the presence and severity of CAD in human angiographic studies [171].

To translate these discoveries into clinical practice, priority should be given to establishing standardized detection assays and protocols for the most promising biomarkers to ensure reproducibility across clinical laboratories. Concurrently, prospective validation of these biomarkers in large, multicenter cohorts should be conducted to rigorously define their predictive value for hard endpoints like incident T2D or major adverse cardiovascular events. While the clinical utility of these biomarkers is supported by human observational data, future mechanistic studies integrating genetic and pharmacological approaches in preclinical models are essential to move beyond correlation and establish causality, thereby unlocking the full therapeutic potential of targeting adipose tissue-derived factors for human diseases.

### Adipose depot-specific gene editing therapy

Recent breakthroughs in gene editing technologies have offered unprecedented opportunities for the precise treatment of metabolic diseases, with increasing recognition of the need to account for adipocyte heterogeneity in therapeutic design. By targeting specific genes within distinct adipose depots, researchers can now intervene with unprecedented cellular and anatomical precision, guided by depot-specific molecular signatures. For example, targeted silencing of the VAT-abundant FABP4 in white adipocytes via the CRISPR interference (CRISPRi) system effectively reduced body weight and inflammation, while also ameliorating hepatic steatosis in obese murine models [172]. The therapeutic efficacy varied depending on the targeted fat depot, with VAT-specific editing producing more pronounced metabolic benefits than SAT-targeted interventions. Additionally, genetic modification of human white adipocytes to acquire brown-like properties represents a valuable route for the development of cell-based therapeutic strategies to treat obesity and diabetes [173]. Consistently, transplantation of CRISPR-engineered human or mouse brown-like adipocytes into high-fat diet-fed mice resulted in reduced adiposity and liver triglyceride levels, along with improved glucose tolerance [174]. Importantly, the metabolic benefits were highly dependent on the engraftment site, with visceral implantation providing superior systemic metabolic improvement compared to subcutaneous placement, further highlighting the critical role of anatomical context in therapeutic efficacy [174]. These advances illustrate the potential of combining biomarker-informed targeting with depot-specific gene editing to develop next-generation therapies for obesity and its related metabolic disorders. However, careful attention must be paid to off-target effects in non-adipose tissues when employing CRISPR-mediated approaches in clinical applications. This can be mitigated by using adipose-specific promoters such as the adiponectin promoter and optimizing CRISPR guide RNA design, strategies that have already been validated in preclinical models and are ready for translational development. Future efforts may integrate single-cell omics analysis of human adipose tissues to identify depot-selective therapeutic targets and refine delivery systems for spatially precise genetic interventions, paving the way for first-in-human studies of depot-specific gene editing for metabolic diseases.

### Precision modulation of targeting depot-specific adipokines

Adipokine-based therapies are emerging as promising strategies for managing metabolic diseases and CVDs, yet their development must consider the heterogeneous nature of adipose tissue, as different fat depots contribute unequally to circulating adipokine levels. Preclinical studies have demonstrated the therapeutic potential of targeting specific adipokine pathways in a depot-specific manner, with interventions showing differential effects based on anatomical and pathological contexts. In metabolic liver disease, agents such as AdipoRon and the active short peptide ADP355 mimicking the effects of SAT-derived adiponectin have shown multiple benefits in preclinical models of MASLD and MASH. These include reductions in hepatic steatosis and fibrosis, improved insulin sensitivity, enhanced mitochondrial function, and attenuated inflammation, positioning them as promising candidates for clinical translation in metabolic liver disease [175]. In cardiovascular contexts, EAT has been identified as a key secretory source of pathogenic mediators. Galectin-3, a profibrotic adipokine enriched in diseased EAT, promotes cardiac fibrosis and atrial fibrillation. Preclinical inhibition of Galectin-3 using modified citrus pectin has been shown to reduce fibrosis and improve cardiac function, highlighting its potential as a therapeutic target specific to EAT-driven pathology [176]. These examples illustrate that therapeutic responses to adipokine modulation are likely influenced by individual variations in adipose tissue distribution and depot activity. Despite these encouraging preclinical outcomes, adipokine-based therapies carry the risk of systemic side effects in clinical trials, which can be minimized by developing adipose depot-selective drug delivery systems (such as SAT- or EAT-targeted nanoparticles) to reduce off-target tissue exposure. Future clinical strategies should integrate imaging and biomarker assessments of fat depot characteristics to enable personalized, depot-specific treatment approaches for metabolic and cardiovascular disorders.

### Therapeutical potential of targeting inflammation in different adipose depots

Adipose tissue inflammation is a key driver of metabolic disease, presenting a promising target for therapeutic intervention. The inflammatory microenvironment varies substantially between VAT and SAT, thus calling for tailored, site-specific strategies. Current approaches primarily focus on modulating ATMs. For instance, in obese mouse models, the sodium-glucose cotransporter 2 inhibitor empagliflozin shifts ATMs from a proinflammatory M1 to an anti-inflammatory M2 phenotype, particularly in VAT where macrophage-driven inflammation is most pronounced [177]. Similarly, genetic silencing of TNF-α converting enzyme in visceral ATMs reduces VAT inflammation and improves systemic glucose control in obese mice [178], while the near-infrared fluorophore IR-61 suppresses ATM activation via mitochondrial reprogramming, mitigating obesity-related metabolic dysfunction in mice [179]. Beyond macrophages, other immune cells offer distinct therapeutic opportunities. VAT-resident Tregs exhibit functional plasticity, tissue-localized expansion, and repertoire diversification under metabolic stress, as characterized in mouse models using single-cell transcriptomics and lineage, positioning them as precise targets for restoring immunometabolic homeostasis [180]. In addition to preclinical findings, clinical evidence supports the therapeutic potential of targeting adipose tissue inflammation. In patients with HF, empagliflozin reduces EAT inflammation and improves myocardial energetics [181]. Similarly, icosapent ethyl decreases EAT volume and inflammatory biomarkers in patients with CVD, with these changes correlating with improved clinical outcomes [182]. Thus, targeting adipose tissue inflammation through depot-specific modulation of diverse immune cells constitutes a sophisticated and evolving strategy for treating obesity-related metabolic diseases, a concept now moving from mechanistic studies in mice toward clinical validation in humans.

### AI-driven imaging analysis of distinct adipose tissues for disease risk prediction

Recent progress in AI has transformed disease risk assessment in humans through the application of adipose tissue imaging and the analysis of quantitative biomarkers. Advanced deep learning models facilitate automated segmentation and detailed characterization of adipose tissue compartments from standard CT and MRI scans, uncovering prognostic information that surpasses conventional measures. These AI approaches are particularly valuable for capturing adipose tissue heterogeneity in patients, enabling precise quantification of different fat depots and their radiological features. Commandeur et al. [183] employed a deep learning algorithm to quantify EAT in coronary CT scans from cardiac patients, demonstrating a strong concordance with expert manual assessments and validating the feasibility of automated EAT quantification in routine clinical workflows. Similarly, a boosted ensemble machine learning approach has been used to evaluate the relationship between EAT volume (derived from noncontrast CT images obtained during PET) and myocardial flow reserve (MFR) in patients undergoing PET/CT [184]. The machine learning-based composite risk score markedly improves the reclassification of individuals with impaired MFR when incorporating EAT volume and coronary calcium score, thereby enhancing risk stratification in clinical settings. Furthermore, a study based on UK Biobank imaging data demonstrates that machine learning-based EAT radiomics phenotyping outperforms traditional area measurements in predicting and detecting HF, enabling earlier personalized intervention in asymptomatic individuals at risk [185]. Radiomic features capturing textural heterogeneity within different fat depots provide additional prognostic information beyond simple volumetric assessments. Recently, several studies using deep learning and genome-wide association meta-analyses or phenome-wide association and Mendelian randomization to analyze bone marrow fat fraction (BMFF) in the UK Biobank have identified the clinical implications of bone marrow adiposity. These studies have revealed the association between BMAT and various diseases, such as osteoporosis, T2D, and CVD, providing population-level evidence for BMAT as a clinically relevant biomarker [186–188]. The deployment of AI-driven adipose tissue analysis is progressively advancing from research settings toward clinical practice, with disease risk prediction emerging as a key near-term application for patient care. These technologies are particularly powerful for characterizing the heterogeneous nature of adipose tissue distribution in individual patients, enabling more precise risk stratification and personalized intervention strategies tailored to each person’s unique fat depot profile.

## Conclusion Remarks and Future Perspectives

Adipose tissue heterogeneity is a core determinant of its dual protective and pathogenic roles in human health and disease, with depot-specific anatomy, cellular composition, and molecular signatures governing systemic metabolism and disease susceptibility. Specifically, VAT emerges as the dominant pathogenic depot, strongly linked to systemic metabolic dysfunction and cardiometabolic diseases. In contrast, SAT acts as a metabolically protective buffer, and thermogenic depots (BAT/beige fat) exert profound beneficial effects on metabolic health. Furthermore, specialized depots like PVAT, EAT, and BMAT function as organ-proximal risk modifiers for specific local pathologies, such as cardiovascular and skeletal/hematological conditions, highlighting that human health is disproportionately influenced by these distinct depot-specific roles rather than by total fat mass alone. Moreover, the intricate cellular composition and lineage heterogeneity within these depots are critical for understanding the variable efficacy of metabolic interventions. Resolving the complex crosstalk between distinct fat depots and other organs has uncovered novel therapeutic avenues with direct relevance to patient care. Understanding the spatial organization and microenvironmental niches is crucial for reconciling seemingly conflicting data and precisely targeting pathogenic mechanisms. Ultimately, realizing the full potential of these discoveries requires translational integration, which can be achieved through advanced imaging, spatial omics, and targeted interventions, ranging from depot-specific gene editing therapy to targeting adipokines and inflammation in different adipose tissues, together with AI-driven risk stratification of metabolic diseases.

Despite substantial progress in this field, several critical challenges persist in translating findings to clinical applications, which require resolutions through advanced research and emerging technologies. First, the molecular mechanisms underlying adipose tissue heterogeneity deduced from animal models must be interpreted cautiously, as species-specific differences between rodents and humans remain underappreciated. This critical gap can be partially addressed using human adipose organoid models, which recapitulate depot-specific traits, intercellular crosstalk, and physiological microenvironments more faithfully than traditional in vitro systems, thereby providing a human-relevant platform for mechanistic studies and drug development. Second, technical challenges remain in isolating rare adipocyte subpopulations such as beige adipocytes and BMAT. Beige adipocytes are scarce, prone to revert to white adipocytes upon stimulus withdrawal, and the identification of functionally distinct subsets has further complicated their isolation [59]. Meanwhile, isolation of BMAT presents unique challenges due to its restricted anatomical distribution and dynamic remodeling in response to metabolic and hormonal cues, coupled with a lack of unique surface markers [44]. Future studies should establish standardized reference for BMAT, including specific cell surface markers and secretome profiles. A third critical bottleneck is the absence of standardized protocols for adipose tissue sampling and spatial mapping. Variations in collection sites, tissue handling, and preservation methods introduce substantial experimental variability [18], underscoring the need for uniform acquisition protocols to ensure reproducibility and comparability across both preclinical and clinical studies. This requires defining specific sampling locations, standardized processing procedures, and consistent storage conditions, while integrating spatial mapping technologies to generate comprehensive adipose tissue atlases. Addressing these challenges will enhance data comparability, improve reproducibility, and accelerate advancements in adipose tissue biology and related metabolic diseases.

**Fig. 4.**
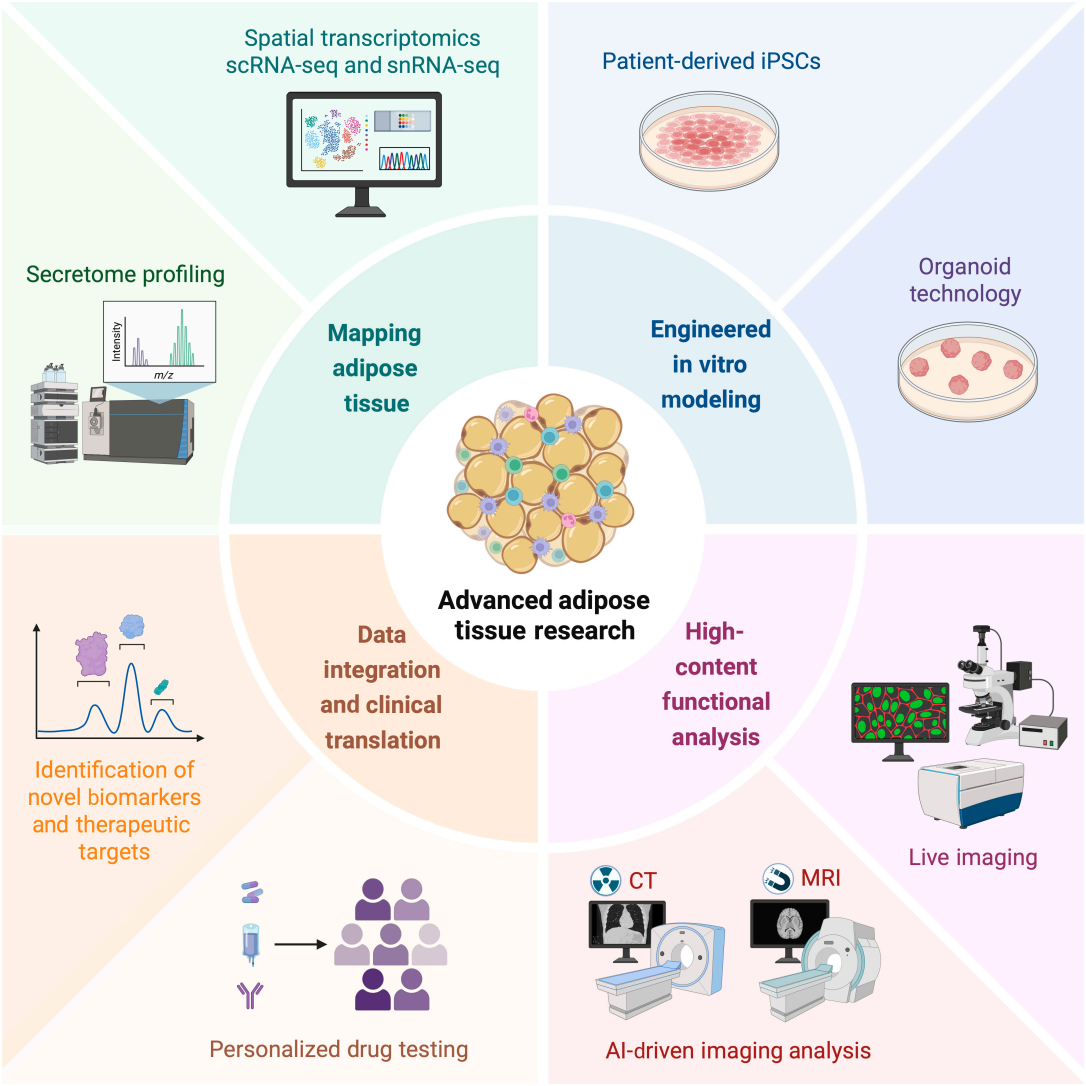
An integrated pipeline for advanced adipose tissue research. Schematic outline of a multiscale workflow to decode adipose depot functional heterogeneity and to accelerate clinical translation. This integrated pipeline combines molecular mapping of adipose tissues via spatial transcriptomics, scRNA-seq and snRNA-seq, engineered in vitro modeling with induced pluripotent stem cells (iPSCs) and organoids, high-content functional characterization through live imaging and AI-driven analysis, and cross-modal data integration to advance biomarker discovery, therapeutic target identification, and personalized drug testing. Illustration created using BioRender (https://BioRender.com).

The future trajectory of adipose tissue research shifts from traditional static, bulk-level analyses toward a dynamic, multiscale comprehension of tissue functionality across various adipose depots to decode their functional heterogeneity and translate insights into personalized clinical care, which rely on integrating spatial transcriptomics, single-cell multiomics, organoid models, and AI tools (Fig. 4). Central to this approach is the synergistic combination of spatial transcriptomics and single-cell multiomics methodologies applied to human adipose specimens. While scRNA-seq and snRNA-seq effectively delineate cellular heterogeneity and uncover regulatory networks, it is worthy to acknowledge that data reproducibility across different laboratories and functional characterization of those novel cell subpopulations remains a major challenge. Additionally, these methods inherently lack the preservation of native tissue architecture. Spatial transcriptomics could address this limitation by providing spatially resolved molecular maps within intact adipose tissue sections, thereby revealing the spatial organization of cellular populations and microenvironments. To truly advance the field beyond descriptive analyses, a more rigorous approach is imperative, emphasizing the urgent need for standardized scRNA-seq and spatial transcriptomics protocols to ensure reproducibility and the integration of functional assays to validate the existence of identified cell types and their biological significance. The integration of spatial transcriptomics, scRNA-seq/snRNA-seq, and secretome profiling in human adipose tissues is expected to facilitate high-resolution mapping of depot- and region-specific cellular states, with a particular focus on characterizing BMAT- and SAT-resident beige adipocytes, both of which exhibit marked regional heterogeneity.

Patient-derived induced pluripotent stem cells (iPSCs) and organoid technologies could recapitulate individual-specific adipose microenvironments, facilitating mechanistic studies of depot-specific dysfunction relevant to human diseases. To maximize their translational impact, these technologies must advance from proof-of-concept models to scalable platforms for testing niche-specific hypotheses with direct clinical applicability. Priority should be given to developing organoids that recapitulate key disease-relevant features of specific adipose depots, such as VAT inflammation in obesity-associated disorders, using cells from patients with well-characterized clinical phenotypes. A key testable milestone involves leveraging these systems for high-throughput screening (such as CRISPRi or small molecules) to identify modifiers of target niches. Complementing these in vitro models, AI-driven imaging analysis and live imaging technology could bridge preclinical observations to in vivo tissue behavior, accelerating the identification of novel biomarkers and therapeutic targets. Building on this, AI-driven analysis of clinical imaging (such as CT and MRI) could be integrated with electronic health records to create a noninvasive “adipose phenotype score” for predicting metabolic and cardiovascular risk in individuals. To ensure clinical translatability, such models must undergo rigorous validation involving retrospective training on well-characterized cohorts with hard clinical endpoints (such as cardiovascular events and diabetes progression), followed by prospective validation of their predictive performance in guiding therapeutic decisions. Combining these AI-derived insights with multiomics and clinical profiles could support personalized drug testing, advance precision strategies, and foster the translation of adipose tissue research into tailored interventions for patients with cardiometabolic disorders. Taken together, these complementary approaches will not only facilitate the unraveling of the full complexity of adipose tissue biology and pathophysiology, but also accelerate the translation from target discovery to the development of novel, efficacious therapeutic strategies for metabolic diseases.
